# Can We Commit Future Managers to Honesty?

**DOI:** 10.3389/fpsyg.2021.701627

**Published:** 2021-08-13

**Authors:** Nicolas Jacquemet, Stéphane Luchini, Julie Rosaz, Jason F. Shogren

**Affiliations:** ^1^Paris School of Economics, Univ. Paris 1 Panthéon-Sorbonne, Paris, France; ^2^Aix Marseille Univ, CNRS, AMSE, Marseille, France; ^3^Univ Lyon, Université Lumière Lyon 2, GATE UMR 5824, Ecully, France; ^4^Department of Economics, University of Wyoming, Laramie, WY, United States

**Keywords:** commitment, lying, In-group bias, managers, honesty, Oath, business ethics, C92, D03, D63.

## Abstract

In a competitive business environment, dishonesty can pay. Self-interested executives and managers can have incentive to shade the truth for personal gain. In response, the business community has considered how to commit these executives and managers to a higher ethical standard. The MBA Oath and the Dutch Bankers Oath are examples of such a commitment device. The question we test herein is whether the oath can be used as an effective form of ethics management for future executives/managers—who for our experiment we recruited from a leading French business school—by actually improving their honesty. Using a classic Sender-Receiver strategic game experiment, we reinforce professional identity by pre-selecting the group to which Receivers belong. This allows us to determine whether taking the oath deters lying among future managers. Our results suggest “yes and no.” We observe that these future executives/managers who took a solemn honesty oath as a Sender were *(a)* significantly more likely to tell the truth when the lie was detrimental to the Receiver, but *(b)* were not more likely to tell the truth when the lie was mutually beneficial to both the Sender and Receiver. A joint product of our design is our ability to measure in-group bias in lying behavior in our population of subjects (comparing behavior of subjects in the same and different business schools). The experiment provides clear evidence of a lack of such bias.

## 1. Introduction

Recent scandals in the business community have raised serious concerns about whether the competitive culture fosters dishonest behavior for the sake of personal profit (see e.g., Cohn et al., 2014). This trepidation has created a growing interest for professional oaths in business (de Bruin, 2016). Observers inside and outside the business community have suggested that future managers, like graduating MBA students, should take a voluntary MBA oath—a commitment to an ethical standard of integrity and honesty (Anderson and Escher, 2010, see, e.g., http://mbaoath.org/). Started in 2001 by a coalition of 2, 000 MBA students from the Harvard Business School, the MBA Oath initiative now covers graduates, advisors and alumni signers from over 500 MBA programs around the world.

But to our knowledge there exists no formal assessment of whether and how a voluntary solemn oath impacts the integrity of future business executives and managers. Building on our recent research on the behavioral impacts of a truth-telling oath (Jacquemet et al., 2017b, 2020, 2021), herein we explore whether future managers respond with more honesty to a voluntary oath that promotes truth-telling. We do so in the context of a laboratory experiment by recruiting students from a renowned business school in France and ask them to perform a classic Sender-Receiver strategic gaming experiment in which dishonesty pays^1^. Each participant sees two rolls of a computerized dice and is asked to communicate the results to another person whose choice will determine the final payoff (see Erat and Gneezy, 2012). We consider two cases: Selfish lies—which are self-beneficial but detrimental to the receiver—and Pareto lies—which are mutually beneficial, i.e., a win-win “white lie.” This game structure allows us to both investigate a rich set of lying behavior thanks to changes in the payoff structure, and to reduce the risk that truth-telling occurs because of sophisticated deception (Sutter, 2009).

Our main treatment variable is a truth-telling oath that participants are offered to sign before they learn the exact nature of the subsequent experiment. The oath procedure has been designed by Jacquemet et al. (2013) in such a way that compliance is voluntary, and most subjects do choose to comply—all subjects do sign the oath in this experiment, while the average is closer to 95% putting together all truth-telling oath experiments that has been carried out over the years. According to accumulated evidence in social psychology, compliance with the oath can commit subjects to truth-telling in subsequent decisions that are aligned with the content of the oath (see, e.g., Joule and Beauvois, 1998; Cialdini, 2007). Jacquemet et al. (2019) show that a requirement for the truth-telling oath to be effective is to remind people when “a lie is a lie” (a condition called “loaded environment”)—because a neutral environment gives subjects more “room to wiggle” and to rationalize lying behavior under oath. They show that, without an oath, selfish lies (resp. Pareto lies) decrease from 41.7% (resp. 68.3%) to 35.7% (resp. 60.0%) when lying is made explicit. In the neutral environment, the oath has no effect on Pareto lies and decreases the proportion of selfish lies to 36.7%. But when lies are made explicit, the oath decreases the proportion of Pareto and selfish lies to 36.7 and 16.7%, respectively. We design our experiment to make lying explicit, and implement the “loaded environment” condition of Jacquemet et al. (2019). This design choice also rules out the possibility that the oath affects truth-telling behavior because the wording of the oath gives subjects a social cue about the appropriate behavior (Haley and Fessler, 2005; Rigdon et al., 2009)^2^.

As a well-targeted subject pool of future executives and managers, we recruited students from a renowned French business school as our oath takers. We assign these future managers to always be in the role of the Sender, and we contrast the lying behavior between future managers under oath to those in a no-oath condition to measure the behavioral effect of a commitment to honesty within this subgroup of population. The obvious challenge to the identification of the effect of professional identity is self-selection into a particular profession, leading to a spurious correlation between behavior and professional identity that goes through unobserved, group-specific, individual heterogeneity. The usual strategy to overcome this issue is to implement exogenous variations in the provision of environmental cues associated with professional identity (Benjamin et al., 2010). According to “self-categorization theory” in social psychology (Turner, 1985), this manipulation makes professional identity more salient and leads subjects to rely more on the norms associated with this identity (this idea that behavior is induced by the norms associated with the identity to which people give more weight due to the circumstances of the choice is at the core of the economics of identity literature initiated by Akerlof and Kranton, 2000, 2010). Following Shih et al. (1999), our instrument to make identity more salient is the group identity of the matched partner: from the same business school or from a different field of study in another school. We purposefully pair our future managers with a partner from either the same business school as the identity inducement condition or from another discipline at another university as the control. This design choice generates data on the effect of the oath on future managers whose professional identity is salient; this feature also provides evidence as to whether an in-group bias drives lying behavior among future managers.

Our results are 3-fold. First, without the oath, future managers lie in both the Pareto and Selfish Lie cases—we observe more dishonesty for Pareto lies (79%) relative to Selfish lies (33%). The magnitude of Selfish lying is similar to that of students in other non-business fields, around 33%. Second, lying is of the same magnitude whether future managers are matched with a peer from the same school or not; we do not observe significant in-group/out-group effects in lying behavior. Third, the oath significantly reduces lying for the Selfish lie case (lying declined by 70%); but the oath had no significant effect on reducing Pareto lies (lies dropped by 14%). This lack of behavioral response, however, does not mean subjects are insensitive to the oath when telling mutually beneficial lies. Using “happiness” as a proxy for subject's internal response, we find that the oath makes lying psychologically more costly—making lying under oath more problematic than without—although not to an extent that is sufficient to change behavior when lying is payoff maximizing for both sides.

## 2. Design of the experiment

The experiment closely follows the extension by Jacquemet et al. (2019) of the sender-receiver game first introduced in Erat and Gneezy (2012). The design relies on three treatment variables: the type of lie (Selfish/Pareto, within-subjects), the group identity (in-group/out-group, between subjects) and the oath (no oath/oath, between subjects), implemented using a 2 × 2 × 2 factorial design.

### Sender-Receiver Game

Two players, a sender and a receiver (labeled ‘player A' and ‘player B' in the written instructions, see the Appendix, section Experimental Instructions, for an English translation of the original instructions in French) are randomly matched. The computer randomly draws a 6-sided die, and informs the sender about the outcome. The sender is then asked to choose between 6 possible messages to send to the receiver: “*The outcome of the roll of die was* [1, 2, .., 5, 6].” Our game replicates the ‘loaded environment' condition of Jacquemet et al. (2019). Accordingly, we explicitly label untruthful communication a “lie” and truthful communication the “truth” in the written instructions. Based on the content of the message, the receiver is asked to choose a number in the set [1, 2, 3, 4, 5, 6], which determines the payment of both subjects between two payment options, X and Y. Only the sender knows the actual payoffs generated by each option. If the number chosen by the receiver matches the die roll, both subjects are paid based on option *X*; otherwise, *Y* is implemented. This is common knowledge to all subjects.

### Types of Lie

Following Erat and Gneezy (2012), we use the combination of payoffs associated with each option as an experimental device to distinguish different types of lies. The payoffs are always set to (20;20) for the sender and the receiver if the sender's choice matches the die roll (option *X*). In the “selfish lie” condition, the payoffs implemented by option *Y* are (21;15): if the sender chooses to lie and the receiver follows the sender's message, the lie imposes a loss of 5 on the receiver, while the sender gains 1. We summarize the type of lie accordingly based on the variation in payoff induced by a lie as *T*[−5;1]. In the Pareto lie, by contrast, both the sender and the receiver benefit from the lie: the payoffs implemented by option *Y* are (30;30), and the lie is accordingly denoted *T*[10;10]. We facilitate inter-study comparison by purposefully selecting the values of the payoff parameters in each treatment to closely follow those used in Erat and Gneezy (2012), and Jacquemet et al. (2019). This choice leads to an asymmetry in the sender's benefit from lying. These two conditions are implemented within subjects in a random order to control for order effects. The roles are fixed, but subjects are randomly rematched with a different subject between the two conditions. To avoid the confounding effect of changes in wealth over the two repetitions of the game, only one condition is binding to determine the actual payment given to subjects at the end of the experiment. Subjects receive no additional information about the other player's decisions or payoffs until the end of the experiment.

### Group Identity

We manipulate group identity within pairs thanks to the school in which participants currently study. The lab is located close to a leading business school, whose students represent a large share (47% as of march 2021) of the lab's subject pool. The master in management offered by this business school lies in the world top-10 according to Quacquarelli Symonds, 2020^3^ ranking and the school itself is part of the 2021 Financial Times top-100 business schools over the world. The experiment focuses on the lying behavior of future managers trained in this school. We always assign the role of sender to a student from this business school. In the in-group treatment (In), the receiver is also a future manager coming from the same business school. In the out-group treatment (Out), the receiver is a student from another school or university, and specialized in a discipline other than Management. These affiliations are made salient on the decision screen for subjects in both roles: once the role has been announced on the screen, a message appears informing participants that “*The player A (B) with whom you will interact studies (**Out**: does not study) at [Name of the business school].”*

### Oath

Before entering the laboratory, each subject is first invited (one by one) to enter an adjacent office. The other subjects could neither hear nor see what happened in the office, as the door was always closed before the start of the procedure. In the No-oath condition while in this office, subjects randomly draw a sheet of paper from an envelope presented to them by the experimenter. The paper indicates the name of the seat they are assigned to in the lab. They are then invited to enter the lab using a side door located between the lab and the office, and the monitor invites the next subject to enter the office.

This no-oath procedure is also applied to receivers in the Oath condition. In contrast, the Senders are exposed to the truth-telling oath procedure designed by Jacquemet et al. (2013). Once they entered the office, subjects are first presented with a form untitled “Solemn oath” (see the Appendix, section English Translation of the Original Oath Form in French, for an English translation of the original form in French). They are asked to read the form and to decide “*freely whether they want to sign it or not*” (the experimenter follows a written script to make sure the subjects are all exposed to exactly the same procedure). The monitor makes clear to subjects that they are free to sign the form, and that neither participation to the experiment nor experimental earnings are conditional on their decision. Whatever their choice, subjects must give the form back to the experimenter, are thanked and invited to draw their seat according to the procedures implemented in the No-oath condition. To avoid communication between subjects prior to the experiment, one monitor stayed in the laboratory during the entire process and helps them find their seat in the room. Subjects receive no information about whether *(i)* other subjects were exposed to the oath procedure, or *(ii)* whether anyone else decided to sign the oath or not^4^.

### Control Variables

A key driver of senders' behavior in our experiment rests in the potential for group-specific attitude toward lying. While senders all belong to the same group, the group of receivers in our experiment can differ by business school, which allow us to measure such heterogeneity. To that end, receivers participate to a simplified (3-sided) version of the dice under the cup task introduced by Fischbacher and Föllmi-Heusi (2013). Subjects then roll a three-sided dice available on their desk. They are told they can roll the dice as many times as they wish, but must report the outcome of the last trial. They are paid for this part according to their report: they earn 0 if they report 1, 1 if they report 2, and 2 if they report 3. Since our aim is to measure *ex ante* heterogeneity, this task is implemented at the start of the experiment, before the sender-receiver game. Senders are not exposed to this task; which allows us to compare their behavior to other experiments using the same sender-receiver game. At the end of the experiment, subjects are asked to fill in several questionnaires aimed at further measuring individual heterogeneity: their level of happiness (7 points Likert scale); their self-reported honesty (7 points Likert scale); the perceived honesty of other subjects (7 points Likert scale); and two measures of cognitive abilities: a 10 items version of the Raven (2008)'s progressive matrices test, and the Cognitive Reflection Test (Frederick, 2005, three reflection questions that must be answered within 60 s). This is followed by Gough (1979)'s Creative Personality Scale (a self-report personality inventory for creativity assessment), which is unrelated to the current paper. Last, we elicit the feeling of closeness to other students based on the “Inclusion of the Other in the Self” scale (IOS, a visual, 7-points, task that measures closeness thanks to the overlap between two circles introduced as representing the other and oneself; Aron et al., 1992; Gächter et al., 2015): subjects are asked about their closeness first with students from the business school and then with students not studying at the business school. The experiment ends with a socio-demographic questionnaire asking participants about their gender, their age, their level of study and the number of times they already participated to an experiment.

### 2.1. Procedures

To implement the group identity treatment variables, subjects were invited separately to the same sessions depending on whether they registered in our subject pool database (managed using HROOT, Bock et al., 2014) as students currently enrolled in the business school or not. We have separate registration lists that allow us to distinguish the group to which subjects belong. Upon arrival, participants are called one by one by their name to check the registration information: they enter the private (oath) office at this stage. The first 10 participants whom we call all are enrolled in the business school list, and are assigned to a computer whose pre-determined role is set to sender. This allows us to control the group identity of senders in all conditions, and to implement the oath procedure on senders only in the Oath conditions.

Once in the laboratory, participants are informed about the instructions of each step of the experiment on their screen (the experiment is computerized using a software developed on Z-tree, Fischbacher, 2007). They can push a button located inside their cubicle to ask a question in private to a monitor at any point in time. All payments in the experiment are expressed in *Experimental Currency Unit*. The exchange rate is 1 ECU = 0.3 euros. Participants are paid a fixed fee for of 5 euros for answering the post-experimental survey, which is added to the payment that results from their decision in the payoff condition of the sender-receiver game that is randomly drawn (and the outcome from the dice-under the cup task for receivers). The average individual payoff is 12.02 euros for an average 1 h participation. We ran 13 sessions (with 260 participants, among whom 130 are senders) of the experiment at the GATE-Lab between September 2019 and February 2020. Table 1 provides the allocation of sessions and participants across the four between-subjects experimental treatments. All 63 senders who participated to an Oathsession agreed to sign the oath. This ensures the behavioral effect of the oath cannot be attributed to self-selection into compliance with the oath request.

**Table 1:** Sample sizes.

	**Total**	**No-oath-Out**	**No-oath-In**	**Oath-Out**	**Oath-In**
Nb. of sessions (senders)	13 (160)	3 (34)	3 (33)	4 (38)	3 (25)

### 2.2. Manipulation Checks

Before moving to the results of the experiment, we use our control variables to provide an overview of the identifying variations induced by the experimental design. The first key dimension in our experiment is to compare interactions of senders with future managers to interactions with non-future managers based on the group identity treatment manipulation. Among the 160 participants who play as a receiver in the experiment, 72 are assigned to the In conditions and are not enrolled in the same business school as senders. The field of study of these Out participants make it highly unlikely they belong to the same group as participants from the target business school: 68% of them are enrolled in one of the two engineering school that are located close to the laboratory. The remaining participants study chemistry, medicine, biology, law, political science and arts. Only three participants study fields that are close to business studies: two in economics, and one in management.

In our design, the manipulation of the group identity of the receiver is instrumental and aims to reinforce the self-identity of senders as future managers. We check the internal validity of the consequences of this group assignment by comparing the answers to the two IOS questions among the senders (following, e.g., Harris et al., 2015). The results unambiguously support that perceived closeness reacts to the treatment manipulation: future managers feel closer to their fellow, with an average closeness equal to 3.96, than to subjects from the other school (3.09, the difference is highly significant, *p* < 0.001, according to paired-sample Wilcoxon rank sum test). This difference prevails whether senders participate to the No-oath (4.13 vs. 3.30, *p* < 0.001) or to the Oath condition (3.78 vs. 2.86, *p* < 0.001).

An important confounding effect in sender-receiver games is the possibility that lying arises as an attempt to counteract the willingness of receivers not to follow the message received (called “sophisticated deception” by Sutter, 2009). To ascertain that senders will not react to treatments because they expect receivers to react differently to their message, we check whether receivers behavior is similar between treatments. Among future managers (In condition) 75.0% of receivers decide to follow the message they receive. This proportion is slightly higher among receivers in the Out conditions, who follow the message 70.1% of the time (*p* = 0.678, χ^2^ bootstrap test)^5^. We observe the same lack of difference in receivers' behavior regarding the implementation of the oath: 69.8% of receivers in Oath and 74.6% of receivers in No-oath decide to follow the message (*p* = 0.736, χ^2^ bootstrap test).

**Figure 1:**
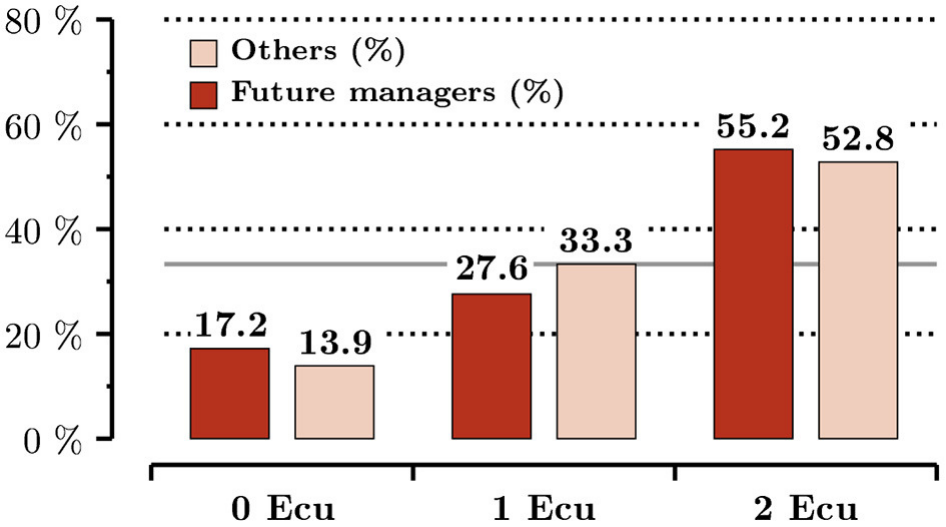
Empirical distribution of dice draws among receivers.

In Figure 1, we provide evidence that group identity does not translate into differences in individual attitudes toward lying, based on the outcomes from the dice under the cup task performed by receivers. We plot the distribution separately within the group of future managers and non-future managers. The horizontal line in gray displays the theoretical benchmark—the uniform distribution that would result from perfectly truthful reports. Among both groups, the empirical distribution of responses clearly departs from the benchmark: the proportion of reported draws that give rise to no earnings is under-represented (*p* = 0.014 for future managers, *p* < 0.001 for other subjects; two-sided proportion test) whereas the report that pays the most is over-represented (*p* < 0.001 and *p* = 0.010; two-sided proportion test). The two almost perfectly balance, as the middle report is in line with the theoretical expectation for both groups (*p* = 0.430 and *p* = 0.607; two-sided proportion test). Importantly, lying behavior is overall similar between future managers and other subjects (*p* = 0.530, two-sided χ^2^ bootstrap test). Under the assumption that receivers' behavior is representative of their group, we conclude that the behavior of senders in our experiment cannot be attributed to group-specific attitudes toward lying.

**Figure 2:**
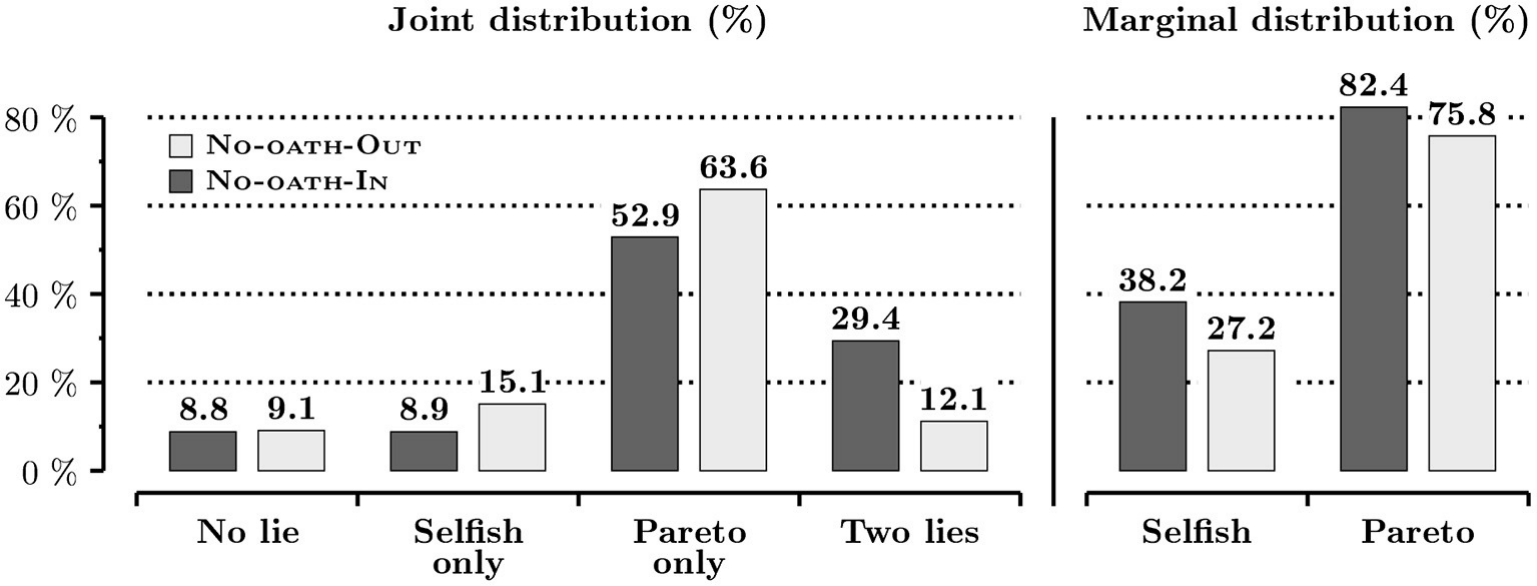
Distributions of lies in the sender-receiver game in the No-oath conditions.

Last, our experiment incidentally provides evidence on in-group-bias in lying behavior by focusing on the No-oath conditions. We observe the induced differences in perceived identity do not translate into different lying behavior in the sender-receiver game. Irrespective of the type of lie, 56.0% of messages sent by future managers in No-oath are dishonest. We observe a small difference in the proportion of dishonest messages between the In and the Out conditions. When future managers send messages to future managers, 51.5% are dishonest as compared to 60.3%. Figure 2 provides a more detailed overview of the pattern of lying in the two conditions. Both the joint distribution of lies over the two games (*p* = 0.731, bootstrap χ^2^ test) and the two marginal distributions of selfish and Pareto lies (*p* = 0.963 and *p* = 0.650; bootstrap proportion tests) are statistically no different across the two No-oath conditions.

## 3. Results

### 3.1. Unconditional Treatment Effects

**Figure 3:**
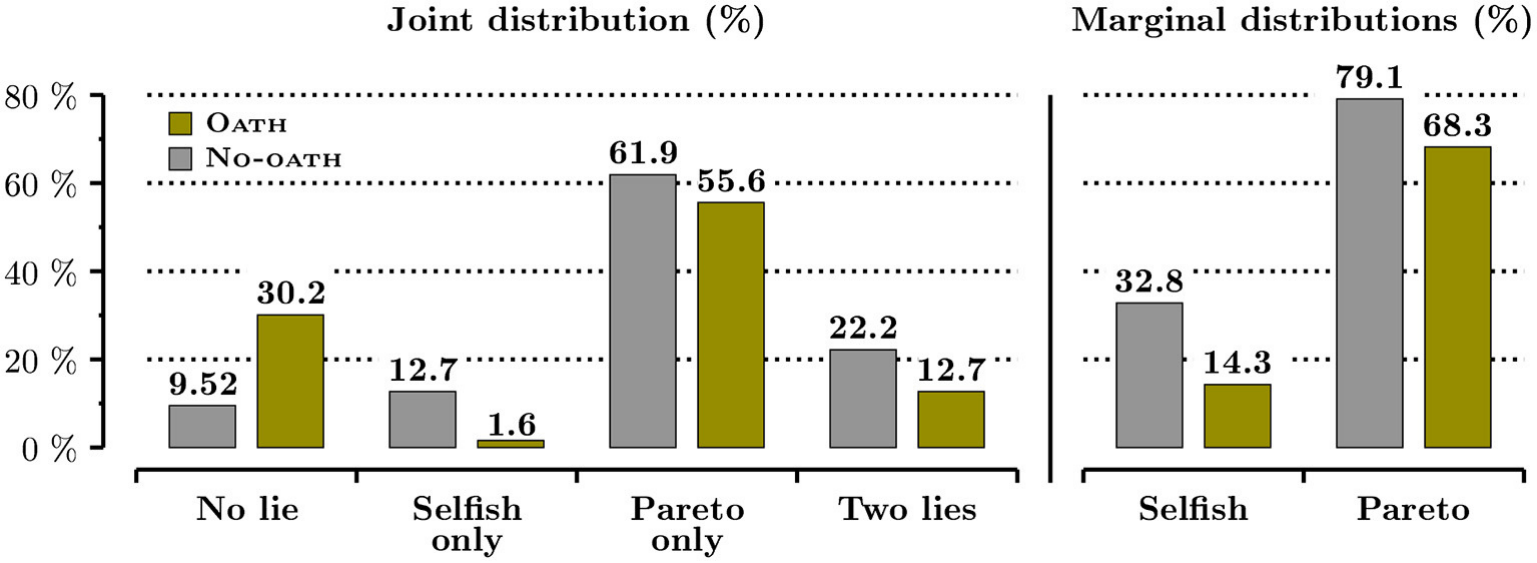
The effect of the oath on the distribution of lying behavior in the sender-receiver game.

We first look at the overall effect of the oath on lying behavior in the sender-receiver game by pooling the two (i.e., In and Out) Oath and the two No-oath conditions. Overall, offering future managers the possibility to sign a truth-telling oath decreases lying by 26.8%. Irrespective of the type of lie, the overall proportion of dishonest messages decreases from 56% in No-oath to 41% in Oath (*p* = 0.070, one-sided bootstrap test). Figure 3 reports the joint and marginal distributions of lying across the two sender-receiver games. The joint distribution clearly shows that the oath induces a drastic increase in the share of fully honest future managers (who send an honest message in both games): their proportion in Oath is more than three times higher (*p* < 0.001, one-sided bootstrap test). This increase in the share of fully honest messages is compensated by a decrease in the share of each one of the three possible patterns of lie. As a results of these sharp differences, the joint distribution is significantly different between Oath and No-oath (*p* = 0.004, bootstrap χ^2^ test). The marginal distributions, displayed on the right-hand side of the figure, indicate that the oath is much more powerful on selfish lies, that happen at the expense of the receiver, than on Pareto lies, that are mutually beneficial to the sender and the receiver. The share of selfish lies is more than twice lower among subjects under oath (*p* = 0.012, one-sided proportion bootstrap test). The slight decrease in Pareto lies is not significant (*p* = 0.153, one-sided proportion bootstrap test), and such behavior remains widespread even under oath.

**Figure 4:**
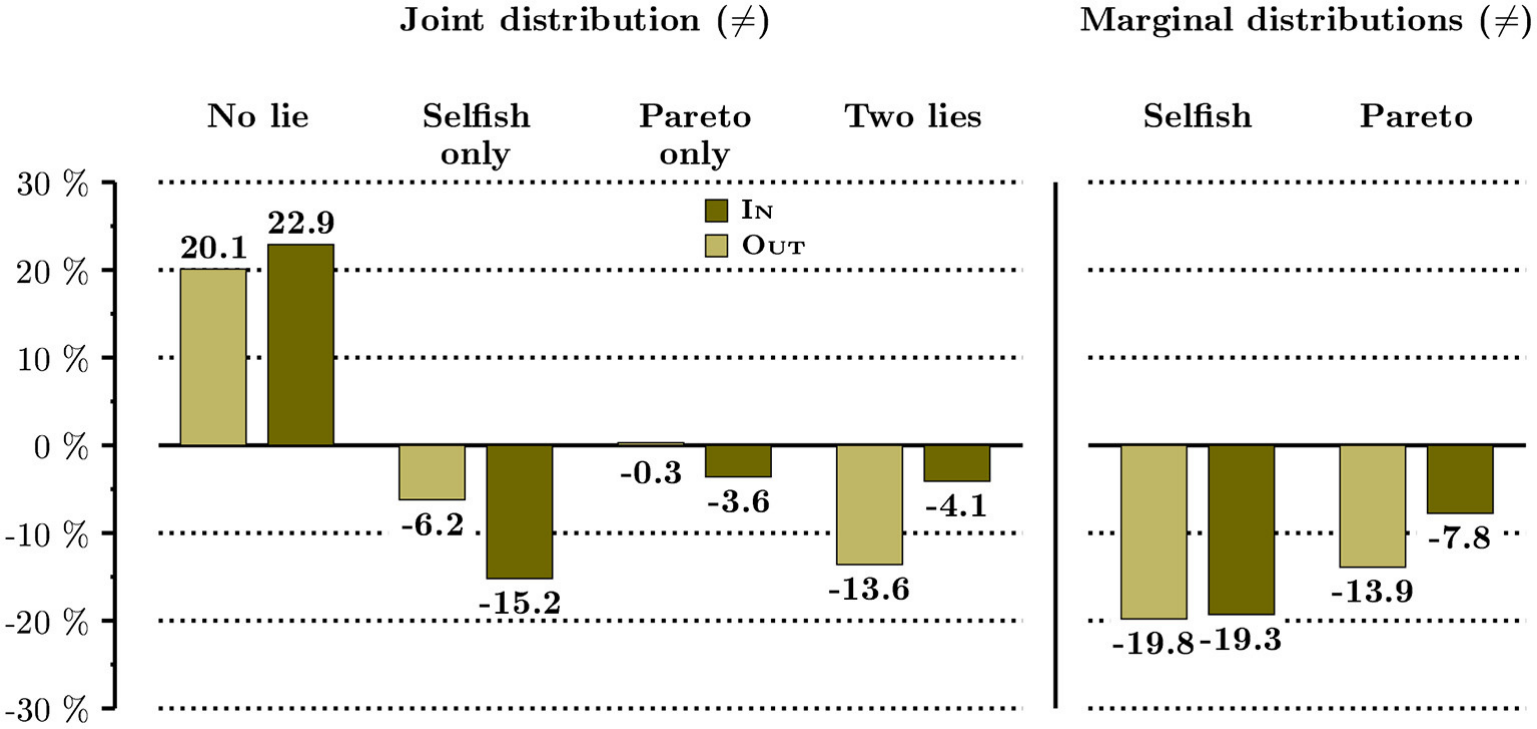
Distributions of the marginal effect of the oath.

In Figure 4, we compare the marginal effect of the oath on lying behavior between In and Out. For each of the two, we report the joint and marginal distributions of the difference in the proportion of lies between No-oath and Oath. The marginal effect of the oath on the share of subjects who decide not to lie is very much alike in the two situations. The main difference rests in how this change is obtained. In Out, full honesty mainly results from a drastic drop in the share of full liars, whereas in In it mainly comes from a decrease in the share of subjects who decide to lie only when this behavior is selfish. This is confirmed by the comparison of the marginal distributions: the decrease in the share of Pareto lies is much higher in Out, while the marginal effect of the oath is similar on selfish lies in both conditions.

### 3.2. Conditional Treatment Effects

We check the robustness of our unconditional results by estimating a multinomial logit model that controls for individual covariates. The dependent variable is lying behavior as defined by the joint distribution: “no lie”, “selfish only”, “Pareto only” and “two lies”. We use honesty (“no lie”) as a reference, so that the coefficients for each of the 3 remaining outcomes can be interpreted in a natural way—a negative sign indicates a decrease in the corresponding lying behavior. We introduce treatment variables and their interactions, as well as subject's age, gender, closeness to other subjects, cognitive abilities (as measured by Raven and CRT scores) and participation to previous experiments.

The estimated parameters, presented in Table 2, confirm that the unconditional results still hold when we introduce subjects' characteristics. First, whether future managers are interacting with a fellow manager or with a student from another field of study has no effect on behavior. In contrast, we also find the oath significantly decreases all types of lying. Results also highlight that observed heterogeneity have very little predictive power on the likelihood that subjects tell a Pareto lie only, or lie in both instances. Selfish lie only stands as a notable exception: subjects who are older and performed better at the Raven test are more likely to engage into this type of lie. On the contrary, subjects who performed better at the CRT test (i.e., who override incorrect intuitive responses and engage in further reflection) are less likely to make a selfish lie only.

**Table 2:** Parametric estimation of the treatment effects.

	**Selfish only**		**Pareto only**		**Two lies**	
	**Parameter**	**p-value**	**Parameter**	**p-value**	**Parameter**	**p-value**
	**estimate**		**estimate**		**estimate**	
Constant	−14.940	0.062	2.771	0.484	−0.424	0.933
Out	0.721	0.571	0.042	0.964	1.201	0.260
Oath×In	−18.696	0.000	−1.661	0.045	−2.020	0.079
Oath×Out	−4.282	0.008	−1.825	0.023	−2.314	0.011
Age	0.622	0.047	0.041	0.822	0.128	0.571
Male	1.485	0.148	0.713	0.180	0.431	0.519
Closeness, BS	−0.357	0.379	−0.305	0.113	−0.198	0.421
Closeness, not BS	−0.190	0.605	−0.209	0.313	−0.432	0.096
Raven score	0.684	0.066	0.111	0.421	0.029	0.870
CRT score	−13.854	0.000	−0.357	0.148	−0.203	0.521
Past experience	−0.339	0.397	−0.238	0.217	0.027	0.907

*Multinomial logit model on the effect of individual characteristics on the likelihood to behave according to one of the four possible lying patterns in the experiment: Selfish lie only, Pareto lie only, or two lies (the reference is honesty in both instances). All explanatory variables are individual specific and do not vary at the individual level; each column reports the estimated effect (along with its p-value) of the corresponding covariate on the likelihood the outcome behavior arises*.

### 3.3. Does the Oath Only Affect Self-Serving Dishonesty?

To sum-up, our experiment provides clear evidence that a truth-telling oath disciplines lying behavior among future managers, but only if lying is detrimental to others. When lying rather serves both the sender's and the receiver's interest, by contrast, we observe very little to no behavioral response to the oath. The obvious question raised by those results is whether lying is perceived as dishonest when it is mutually beneficial, and if yes why we do not observe the same drastic decrease as when lying is selfish.

**Figure 5:**
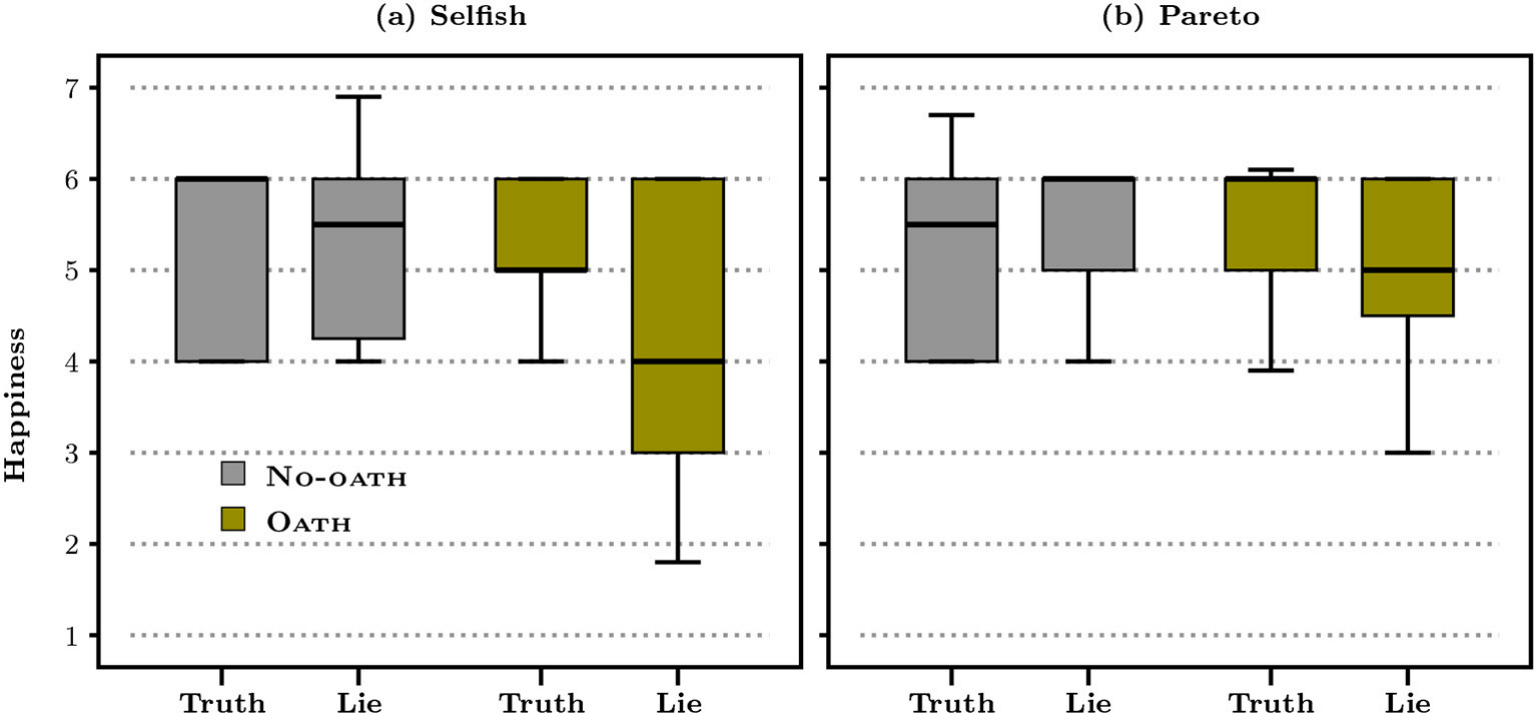
Happiness by treatment and type of lie.

To answer this question, we use the self-reported level of happiness collected at the end of the survey as a proxy of the internal response of subjects to the oath (see e.g., Clark, 2015, for a discussion of the internal validity of self-reported well-being as a mesaure of individual well-being). Since the level of happiness itself is difficult to interpret, we focus on variations between responses (see Jacquemet et al., 2017a, for a similar approach) and focus in Figure 5 on the level of self-reported happiness among senders who truthfully report the outcome of the dice (“truth”) and those who lie, separately by treatment. This boxplot displays the interquartile range, i.e., the distance between the upper (75th percentile) and lower (25th percentile) quartiles. Wiskers present the 10th percentile on the bottom and the90th percentile on the top end. The bold horizontal line displays the median.

Focusing on No-oath subjects, we find no change in the median level of happiness according to lying behavior in both the selfish lie (the median level of happiness among liars is 5.5 vs. 6 among truth-tellers; *p* = 0.280, bootstrap KS test) and the Pareto lie (6 vs. 5.5; *p* = 0.710, bootstrap KS test) situations. Under oath, by contrast, lying comes with a sharp shift in happiness as compared to truth-telling for both kinds of lies: the median level of happiness is lower among liars as compared to truth-tellers in the context of both selfish lies (4 vs. 5; *p* = 0.026, KS bootstrap test) and Pareto lies (5 vs. 6; *p* = 0.006, KS bootstrap test). Median happiness reaches its lowest level, equal to 3.5, among subjects who engage in both a selfish and a Pareto lie (*p* = 0.013 as compared to happiness among subjects who engage in Pareto lies only; KS bootstrap test)^6^. We observe a strong internal response of subjects to the oath when they decide to lie, whatever the lying situation. This response suggests that the oath makes lying psychologically more costly. The lack of behavioral response to the oath in the Pareto lie situation suggests that the benefits of a Pareto lie still outweigh the cost of lying under oath.

## 4. Conclusion

Despite a disappearance of occupational oaths at the end of the 20th Century (Prodi, 1992), the idea of the oath has gained renewed momentum in recent years following the economic crisis and recent business scandals as a form of ethics management. In this paper, we provide the first experimental evidence of the efficacy of the oath to foster integrity of future managers. Our measure of integrity is lying behavior in two classic sender-receiver games, one mutually beneficial (win-win white lies) and one self-serving (selfish lies), in which senders are recruited from the same leading business school. In our experimental design, we vary the group to which the receiver belongs so we can test how the strength of one's professional identity (in-group or out-group) affects the behavior of our subjects.

Our baseline framework (no honesty oath) leads to several useful findings. First, in contrast with what has been observed among criminals (Cohn et al., 2015), we do not find any effect of the professional identity of managers on their lying behavior. Second, thanks to the instrumental manipulation of the group matching of subjects, our study also contributes to the burgeoning literature about in-group biases in lying behavior. The existing evidence that relies either on the minimal group paradigm (Tajfel, 1970), or on natural identities, is mixed. For example, Butler (2014) finds reduced in-group lying when identity is artificial and Maximiano and Chakravarty (2016) find a similar result with natural identities. In contrast, our results are aligned with those from the study that is closest to ours: using natural identities (based on university enrollment), Feldhaus and Mans (2014) do not find any difference in lying behavior in a sender-receiver game between in- and out-group interactions (examples of null results using the minimal identity paradigm include Benistant and Villeval, 2019; Casoria et al., 2020). While we replicate these results and confirm their robustness, we also add control variables allowing to measure both the intensity of the group manipulation and the attitudes toward lying among groups. Despite a significant change in perceived closeness when interactions happen within groups, we confirm the lack of difference between in-group and out-group interactions. Importantly, this happens in a context in which attitudes toward lying are no different between groups.

For both these in-group and the out-group conditions, our main treatment variable of interest is a truth-telling oath that senders are free to sign—and which all subjects do agree to sign. Overall, our results suggest that a solemn oath like the MBA oath can increase the honesty of our future managers when the lie is for selfish reasons. The oath was less powerful on future managers, however, in reducing the frequency of “white lies” or win-win lies. This departs from previous evidence about the truth-telling oath obtained in the same setting but with students from other disciplines. Specifically, Jacquemet et al. (2019) show that (i) Pareto lies are less widespread than with future managers (60.0 vs. 79.1% herein); (ii) strongly react to the oath, with a share of Pareto lies under oath equal to 36.1% (as compared to 68.3%). Although the behavioral responses are drastically different, our results suggest that future managers do react to the oath even in the Pareto lie condition: self-reported happiness data show that the oath makes Pareto lies psychologically more costly, although not to an extent that is sufficient to undermine win-win lying behavior. An important difference between managers and the lay public is the rise in the “win-win" culture, a paradigm that promotes the alignment of interest of stakeholders, in business education and practice (see e.g., Cook, 2017, for a discussion and a historical perspective). We speculate that managers face more salient conflicting motivations when lying is mutually beneficial — an issue we leave for future research.

## Data Availability Statement

The raw data supporting the conclusions of this article will be made available by the authors, without undue reservation.

## Ethics Statement

The studies involving human participants were reviewed and approved by GATE-Lab Review Board for ethical standards in research-Groupe d'Analyse et de Theorie Economique (GATE UMR CNRS 5824). The patients/participants provided their written informed consent to participate in this study.

## Author Contributions

All authors listed have made a substantial, direct and intellectual contribution to the work, and approved it for publication.

## Conflict of Interest

The authors declare that the research was conducted in the absence of any commercial or financial relationships that could be construed as a potential conflict of interest.

## Publisher's Note

All claims expressed in this article are solely those of the authors and do not necessarily represent those of their affiliated organizations, or those of the publisher, the editors and the reviewers. Any product that may be evaluated in this article, or claim that may be made by its manufacturer, is not guaranteed or endorsed by the publisher.

## Footnotes

^1^While the control over the environment offered by a laboratory experiment is better suited to testbed the effect of the oath on future managers, it raises the obvious concern of whether these results would extend to a non-laboratory setting. On this issue, we refer the reader to the literature that correlates lying in lab experiments to unethical behavior in the field (e.g., Potters and Stoop, 2016; Dai et al., 2017; Hanna and Wang, 2017; Cingl and Korbel, 2020) and the ones that investigate both truth-telling behavior under oath in the context of field experiments (Carlsson et al., 2013; Koessler et al., 2019; Jacquemet et al., 2021) ot the long-lasting effects of promises (Peer and Feldman, 2021).

^2^An alternative interpretation of the effect of the oath is the idea that it simply primes subjects to truth-telling. Such an effect is not only unlikely based on the existing literature (Pashler et al., 2013), but is also ruled out by this design choice since the rules of the game themselves make an explicit reference to lying.

^3^ https://www.qs.com/

^4^An obvious methodological concern with.

^5^We test the differences between treatments at the individual level to account for potential within-subject correlation in receivers' behavior across the two lying conditions.

^6^We cannot compare this figure to the level of happiness of subjects who only engage in a selfish lie as only one subject does so in the oath treatment.
