# Supplementary material for: Can We Commit Future Managers to Honesty?
Source: Front Psychol. 2021 Aug 13;12:701627. doi: 10.3389/fpsyg.2021.701627 (PMC8414966; doi:10.3389/fpsyg.2021.701627)
Supplement: Supplementary file 1 [file Data_Sheet_1.pdf]

Can we commit future managers to honesty?

Appendix

A Experimental instructions

Since the experiment proceeds differently for senders (player A) and receivers (player B), participants are informed about the instructions on their screen and go trough the text by themselves. They must confirm they read the instructions at the end of each screen, and cannot move back and forth in the instructions. We provide below a screen by screen English translation of the original instructions in French; the players who see the corresponding screen appear in bracket next to the screen number. We present the instructions for the IN conditions. Variations implemented in the OUT condition appear in bracket and in italic. The name of the target business school has been anonymized and replaced by "BS".

[Screen 1 — A&B]

☐ I study at "BS"
☐ I do not study at "BS"

[Screen 2 — A&B]

This experiment aims to analyze how people make decisions. We ask you to pay close attention to the instructions. They should help you fully understand the experiment.
All of your decisions will be processed anonymously. You will enter your choices on the computer in front of you. Throughout the experiment, you will accumulate gains in ECU. These gains will be converted to Euros (1 ECU = 0,3 euros) at the end of the experiment, and paid in cash. From now on, we ask you not to communicate. Should you have any question, please raise your hand, a staff member will answer you in private.

[Screen 3 — B only]

You must throw the dice that is contained in the cup on your desk by stirring the cup well (and without removing the lid). Your task is to report the color that appears on the top of the dice after looking through the lid.
Note that, if you wish, you can roll the dice more than once. However, you only have to report the result of your first throw.
Your earnings for this game depends on the result of the dice roll: Blue gives 0 ECU, Yellow gives 1 ECU, Red gives 2 ECU.

[Screen 4 — A&B]

In this experiment, players will be divided into two types: type A and type B. You will be informed of your type as the experiment starts. This type will stay the same throughout the experiment.
This experiment contains 2 periods.At each period, pairs are formed by matching an A-type player with a B-type player. The pairs change at each period. Two participants A and B cannot be in the same pair twice during the experiment.
You will have no way of identifying the other member of your pair, and vice versa. However, at each period, the computer will tell you whether or not the other participant in your pair is studying at "BS".

[Screen 5 — A&B]

Each period has two steps.

Step 1: Information and decision-making for player A

Player A is first informed of the payoffs for two possible options: option X and option Y. These payoffs change from one period to another.
Player A must then roll a six sided dice, and is the only participant to know the outcome of this roll.
Once informed of the dice rolls' outcome, player A must choose a message to send to player B.
Player A has a choice between: (1) being honest and telling the truth about the dice roll's outcome; or (2) lying and sending a message that is different from the real outcome.
Please note that player B is only shown player A's message: if player A chooses to lie (choice no2), player B has no way of knowing it.
The message sent to player B is chosen among the following:

- message 1: the outcome of the dice roll is 1;
- message 2: the outcome of the dice roll is 2;
- message 3: the outcome of the dice roll is 3;
- message 4: the outcome of the dice roll is 4;
- message 5: the outcome of the dice roll is 5;
- message 6: the outcome of the dice roll is 6.

[Screen 6 — A&B]

Step 2: information and decision-making for player B

Once all of the A-type players have finished step 1, each B-type player is informed of the message sent by the A-type player they are paired up with.
After reading the message, player B must choose a number between 1 and 6. Their only information is that contained in player A' message: they do not know the real outcome of the dice roll.
The number chosen by player B determines the gains of players A and B. There are two options

concerning the gains: option X and option Y. These options were only described to player A at the beginning of the period.
Each option is associated to specific payoffs for player A and player B.
If the number chosen by player B is identical to the dice outcome, then the payoffs for both players of the pair will be those described in option X.
If the number chosen by player B is different from the dice outcome, then the payoffs for both players of the pair will be those described in option Y.

At the end of the two periods, a screen will summarize the 2 periods along with the related payoffs in ECU. One of the two periods will be randomly chosen for the payment of your gains. Your gains for the experiment will be those earned during that period. These gains will be converted to Euros according to the following exchange rate: 1 ECU=0.3 Euros.

[Screen 7 — A&B]

You are player A(B).

The player B(A) with whom you are paired studies at “BS” (OUT*: does not study at “BS”*).

[Screen 8 — A]

You are player A.

The player B with whom you are paired studies at “BS” (OUT*: does not study at “BS”*).

The payoffs associated with the two possible options are :

Option X : 20 ECU for you, 20 ECU for player B
Option Y : 30 (*Selfish: 21*) ECU for you, 30 (*Selfish:15*) ECU for player B.

The outcome of the dice roll is XXX

If the number chosen by player B is identical to the dice outcome, then the payoffs for both players of the pair will be those described in option X.
If the number chosen by player B is different from the dice outcome, then the payoffs for both players of the pair will be those described in option Y.

Which message would like to send to player B?

☐ The outcome of the dice roll is 1
☐ The outcome of the dice roll is 2
☐ The outcome of the dice roll is 3
☐ The outcome of the dice roll is 4
☐ The outcome of the dice roll is 5
☐ The outcome of the dice roll is 6

[Screen 9 — B]

You are a player B.

The player A with whom you are paired studies at “BS”.

The message sent by player A is

"The outcome of the dice roll is XXX"

Which number do you choose?

☐ The outcome of the dice roll is 1
☐ The outcome of the dice roll is 2
☐ The outcome of the dice roll is 3
☐ The outcome of the dice roll is 4
☐ The outcome of the dice roll is 5
☐ The outcome of the dice roll is 6

[Screen 10 — A&B]

Period 1 is over.

Period 2 will start. You will be paired with another player.

[Screens 11 to 14 replicate screens 7 to 10, with the alternative payoff option]

[Screen 15 — A]

In the first period, when you made your decision about which message to send to player B, would you say your decision was ?
☐ 1 = Totally uncertain
☐ ...
☐ 7 = Totally certain

[Screen 16 — A]

In the second period, when you made your decision about which message to send to player B, would you say your decision was ?
☐ 1 = Totally uncertain
☐ ...
☐ 7 = Totally certain

[Screen 17 — A&B]

We are now asking you to complete various questionnaires.
You will receive 5 euros which will be added to your earnings to compensate you for your travel and for the time spent answering these questionnaires.
Before receiving your earnings from the experiment, please answer a series of questions. There are no right or wrong answers, take the time to think about each of the answers you give because your answers are important to the research you are involved in. We remind that all your decisions are anonymous.

[Screen 18 — A&B]

Using the scale below, indicate how happy you are currently:
☐ 1 = Totally sad
☐ ...
☐ 7 = Totally happy

[Screen 19 — A&B]

Using the scale below, indicate how honest you were during the experience:
☐ 1 = Not honest at all
☐ ...
☐ 7 = Totally honest

[Screen 20 — A&B]

Using the scale below, indicate how honest the other players were during the experience:
☐ 1 = Not honest at all
☐ ...
☐ 7 = Totally honest

[Screen 21 — A&B]

We now ask you to participate to a Raven test.
This test is frequently used to measure intelligence through Intellectual Quotient (IQ). It has often been observed that performance on this test is correlated with academic success and future earnings.
On the next screen you will see an example of the type of questions contained in the test. The solution is indicated in red on the right of the screen.

[Screen 22 — A&B]

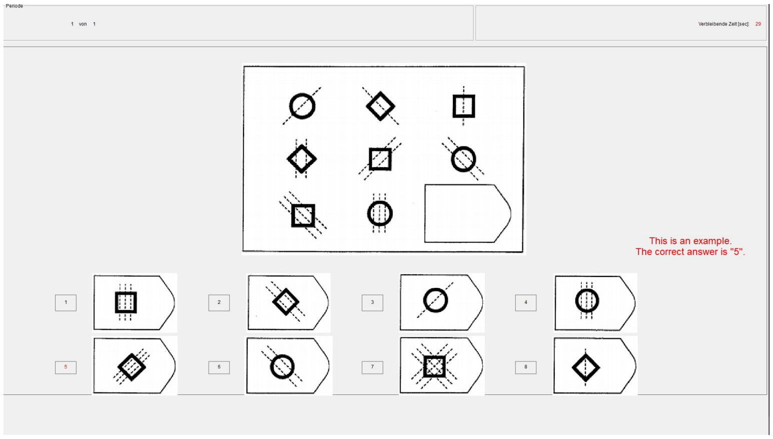

[Screen 23 — A&B]

You will now take the Raven test.
It consists of 10 questions. You will have 600 seconds to answer the 10 questions.

[10 screens for the Raven test]

[Screen 34 — A&B]

A new questionnaire will soon appear on your screen.
The response time for this questionnaire is very short: 60 seconds.
The remaining time will be shown on the screen.

[Screen 35 — A&B]

(1) The cost for a notebook and a pencil is 1.10 Euros. The price of a notebook is 1 Euro more than that of thee pencil. How much does the pencil cost? Answer (cents)

(2) Assuming that it takes 5 machines 5 minutes to manufacture 5 pens, how long would it take 100 machines to manufacture 100 pens? Answer (minutes)

(3) A bank of water lilies grows on a pond. The size of the bank doubles each day. Assuming it takes 48 days for the bank to cover the entire pond, how long would it take for the bank to cover half the pond? Response (days)

Once you have answered all questions, please press OK.

[Screen 36 — A&B]

Check the adjectives in the list below that best describe you. [*Gough (1979)'s list of adjectives*]

[Screen 37 — A&B]

In the following figure, we ask you to choose the circle pair that best indicates how close you feel to students enrolled at "BS".

[Screen 38 — A&B]

In the following figure, we ask you to choose the circle pair that best indicates how close you feel to the students not enrolled at "BS".

[Screen 39 — A&B]

Your earnings for this experiment :
The period X was randomly selected for payment.
Your earnings for this period was XX ECU, so XX euros.
You earned 5euros more as a show-up fees and for the time spent answering the different questionnaires.
In total, your earnings are XXX euros.

[Screen 40 — A&B]

How old are you? ____________________

What is your gender? ☐ Male ☐ Female

In which school year are you? ☐ 1 ☐ 2 ☐ 3 ☐ Beyond

How many experiments have you participated in before this one? ____________________

B English translation of the original oath form in French

In the OATH conditions, the form below is given to subjects once they entered the private office. The experimenter does not describe the form, but asks subjects to carefully read it.

GATE
Lyon / St-Etienne

cnrs

SOLEMN OATH

I, the undersigned ____________________ swear upon my honnour that, during the whole experiment, I will:

Tell the truth and always provide honest answers.

Eccully, ____________________

Signature

Groupe d'Analyse et de Théorie Économique, 93, chemin des Mouilles 69130 Ecully - FRANCE
